# Characterization of *Halomonas* sp. ZM3 isolated from the Zelazny Most post-flotation waste reservoir, with a special focus on its mobile DNA

**DOI:** 10.1186/1471-2180-13-59

**Published:** 2013-03-14

**Authors:** Lukasz Dziewit, Adam Pyzik, Renata Matlakowska, Jadwiga Baj, Magdalena Szuplewska, Dariusz Bartosik

**Affiliations:** 1 University of Warsaw, Faculty of Biology, Institute of Microbiology, Department of Bacterial Genetics, Miecznikowa 1, Warsaw, 02-096, Poland; 2 University of Warsaw, Faculty of Biology, Laboratory of Environmental Pollution Analysis, Miecznikowa 1, Warsaw, 02-096, Poland

**Keywords:** *Halomonas* sp., Mobile genetic elements, IncU plasmid, Transposable elements, Heavy metal resistance, Bacterial adaptation

## Abstract

**Background:**

*Halomonas* sp. ZM3 was isolated from Zelazny Most post-flotation mineral waste repository (Poland), which is highly contaminated with heavy metals and various organic compounds. Mobile DNA of the strain (i.e. plasmids and transposons) were analyzed in order to identify genetic information enabling adaptation of the bacterium to the harsh environmental conditions.

**Results:**

The analysis revealed that ZM3 carries plasmid pZM3H1 (31,370 bp), whose replication system may be considered as an archetype of a novel subgroup of IncU-like replicons. pZM3H1 is a narrow host range, mobilizable plasmid (encodes a relaxase of the MOB_V_ family) containing mercury resistance operon (*mer*) and *czcD* genes (mediate resistance to zinc and cobalt), which are part of a large truncated Tn*3* family transposon. Further analysis demonstrated that the phenotypes determined by the pZM3H1 resistance cassette are highly dependent on the host strain. In another strand of the study, the trap plasmid pMAT1 was employed to identify functional transposable elements of *Halomonas* sp. ZM3. Using the *sacB* positive selection strategy two insertion sequences were identified: IS*Hsp1* - representing IS*5* group of IS*5* family and IS*Hsp2* - a distinct member of the I*S630* family.

**Conclusions:**

This study provides the first detailed description of mobile DNA in a member of the family *Halomonadaceae*. The identified IncU plasmid pZM3H1 confers resistance phenotypes enabling adaptation of the host strain to the Zelazny Most environment. The extended comparative analysis has shed light on the distribution of related IncU plasmids among bacteria, which, in many cases, reflects the frequency and direction of horizontal gene transfer events. Our results also identify plasmid-encoded modules, which may form the basis of novel shuttle vectors, specific for this group of halophilic bacteria.

## Background

The Zelazny Most surface waste management system is the largest mineral waste repository in Europe and one of the largest in the world. It is located in the Lubin-Glogow Copper District in southwest Poland and covers an area of 13.94 km^2^. Polymetallic organic-rich copper ore is currently mined underground in this area. This ore is characterized by its neutral or slightly alkaline pH (of up to 8.5) and its high salinity.

Zelazny Most reservoir was built in 1974 to collect flotation tailings from three local copper-ore enrichment facilities, for the storage of groundwater from the Lubin-Glogow mines, and to be used to facilitate flotation of sulfides during ore processing and transport of the gangue. The total volumes of wastes and water present in Zelazny Most are estimated to be 476 mln m^3^ and 7.5 mln m^3^, respectively. The annual deposition of flotation tailings varies from 20 to 26 million tons [1].

The deposits in Zelazny Most have an alkaline pH (8.5) and are highly contaminated with heavy metals (Cu, Pb, As, Ni, Co, Zn and Cr) and various organic compounds, including polycyclic aromatic hydrocarbons (PAH) such as anthracene, biphenyl, dibenzofurane, dibenzothiophene, chrysene, fluoranthene, fluorene, naphthalene, methylnaphthalene, methylphenanthrene, phenanthrene and pyrene ( [2] and unpublished data). Zelazny Most is located in a seismically active area; however the seismicity is not a natural phenomenon, but is induced by the mining works in the nearby underground copper mines. This seismic activity could lead to the release of the contents of Zelazny Most to the environment, which would have devastating consequences [3].

The water stored in Zelazny Most is of the Cl-SO_4_-Na-Ca type with mineralization levels of up to 21,400 mg l^-1^. The respective concentrations of sodium (Na^+^) and chlorine (Cl^-^) ions are up to 4500 mg l^-1^ and around 8000 mg l^-1^, which makes this environment extremely salty [4]. Saline environments are inhabited by specialized microorganisms, typically halophilic *Archaea* (e.g. *Halobacteriaceae*) and *Bacteria* (e.g. *Halomonadaceae*).

The family *Halomonadaceae* (*Oceanospirillales*, *Gammaproteobacteria*) currently is comprised of 9 genera. These are chemoorganoheterotrophic, aerobic or facultatively anaerobic bacteria, most of which are halophilic or halotolerant. The genus *Halomonas* (type species *H. elongata*, isolated in 1980) contains over forty named species. Members of the *Halomonas* genus have a respiratory-type metabolism with oxygen as the terminal electron acceptor, although some can use nitrate as an alternative acceptor under anaerobic growth conditions. These bacteria are prototrophs able to utilize a large range of organic compounds as their sole carbon and energy source (e.g. carbohydrates, amino acids, polyols, hydrocarbons). The majority of them require Na^+^ ions for growth (0.1-0.3%) and all can grow in a broad range of NaCl concentrations (0.1-32.5%) [5]. Halomonads may be isolated from various saline environments, regardless of their geographical location (e.g. marine environments, saline lakes and soils, intertidal estuaries, solar salt facilities, salty foods). Four species were isolated from the rhizosphere of xerophytic plants [6].

Extreme halophiles, including halomonads, are sources of a variety of bioproducts that can function under conditions of high salt: (i) compatible solutes that have a stabilizing and protective effect on biomolecules, cell structures and whole cells, (ii) extracellular enzymes adapted to saline stress, (iii) biosurfactants, (iv) extracellular polysaccharides and (v) poly-β-hydroxyalcanoates. The use of halophiles in the production of these compounds can significantly lower the cost of fermentation and recovery processes, since high salt concentrations reduce the possibility of contamination by non-halophilic microorganisms, thus, the energy requirement for sterilization can be significantly decreased [7,8].

In recent years, several *Halomonas* spp. genomic projects were initiated, but so far only the genome of the ectoine producer *Halomonas elongata* DSM 2581 has been completed [9]. Current knowledge of mobile genetic elements (MGEs) of halomonads is also very poor. Several *Halomonas* spp. plasmids have been described, but only the narrow-host-range (NHR), mobilizable, cryptic plasmid pHE1 (4.2 kb) of the moderately halophilic bacterium *H. elongata* ATCC 33174 has been characterized in detail [10,11]. In addition, a temperate phage PhiHAP-1, which possesses a linear plasmid-like prophage genome, was isolated from *Halomonas aquamarina* and sequenced [12].

In this study, we have analyzed strain *Halomonas* sp. ZM3, isolated from Zelazny Most during the Bioshale project (a part of this project was to identify microbiological consortia useful in mineral processing) [13]. We have performed complex structural and functional analyses of mobile genetic elements of this strain, specifically plasmid pZM3H1, responsible for adaptation of the host strain to the harsh environment and two insertion sequences (ISs) captured using the trap plasmid pMAT1. To our knowledge this is the first description of functional transposable elements in halomonads.

## Methods

### Bacterial strains, plasmids and culture conditions

The strain ZM3 was isolated from a sample of the flotation tailings of Zelazny Most (Poland). The sample (10 g) was resuspended in 20 ml of sterile salt solution (0.85% NaCl), shaken at 22°C for 2 h and streaked onto the solid LB medium. Plates were incubated at 22°C for 1–2 weeks. The isolated strain was classified as a member of the *Halomonas* genus by 16S rDNA sequence similarity. Other bacterial strains used in this study were (i) *Eschericha coli* TG1 [14], (ii) *E. coli* BR825 [15], (iii) *Agrobacterium tumefaciens* LBA288 [16], (iv) *Paracoccus versutus* UW225 [17], (v-xv) *Alcaligenes* sp. LM16R, *Halomonas* sp. ZM3R, *Pseudomonas* spp. – strains LM5R, LM6R, LM7R, LM8R, LM11R, LM12R, LM13R, LM14R, LM15R (rifampin resistant derivatives of wild-type strains isolated from Lubin copper mine).

The following plasmid vectors were used: (i) pABW1 (Km^r^; *ori* pMB1; *oriT* RK2) [18], (ii) pBBR1-MCS2 (Km^r^; *ori* pBBR1; broad-host-range cloning vector; *oriT* RK2) [19] and (iii) pMAT1 (Km^r^; *ori* pBBR1; *oriT* RK2; *sacB*; trap plasmid) [20]. Plasmids constructed in this work were: (i) pABW-ZM3H1 (Km^r^; *ori* pMB1; *ori* pZM3H1; *oriT* RK2) – mobilizable *E. coli*-*Halomonas* spp. shuttle plasmid constructed by insertion of an EcoRV restriction fragment of pZM3H1 (containing the plasmid replication system) into the BamHI site of pABW1 (BamHI 5' overhangs filled with Klenow fragment of DNA polymerase I), and (ii) pBBR-ZM3CZCMER (Km^r^; *ori* pBBR1; *oriT* RK2) – EcoRI-NheI restriction fragment of pZM3H1, containing resistance determinants, inserted between the SmaI and EcoRI sites of pBBR1MCS-2 (NheI 5' overhang filled with Klenow fragment of DNA polymerase I).

Bacterial strains were grown in LB (lysogeny broth) medium [21] or mineral basal salts (MBS) medium [22] at 37°C (*E. coli*) or 30°C (other strains). Where necessary, the medium was supplemented with kanamycin (50 μg/ml), rifampin (50 μg/ml) and sucrose (10%).

### Temperature, pH and salinity tolerance analyses

The temperature, pH and salinity tolerance of *Halomonas* sp. ZM3 were analyzed by monitoring changes in optical density (in comparison with non-inoculated controls) during incubation of cultures in titration plates, with the aid of an automated microplate reader (Sunrise, TECAN). Overnight cultures were diluted in fresh LB media with adjustments for the separate assays: (i) pH 7.0 for the temperature tolerance analysis, (ii) pH 2.0-13.0 for the pH tolerance analysis, or (iii) supplemented with NaCl to final concentrations of 0.5%, 3%, 6%, 9%, 12% or 15%. In each case, the initial optical density at 600 nm (OD_600_) was 0.05. The microplates were then incubated with shaking at 30°C (for pH and salinity tolerance analysis) or 4°C, 15°C, 22°C, 25°C, 30°C, 37°C, 42°C or 50°C (for temperature tolerance analysis) for 48 hours.

### Utilization of polycyclic aromatic hydrocarbons

To test the ability of bacterial strains to utilize anthracene, phenanthrene, fluoranthene, fluorene and pyrene, the modified PAH plate assay was employed [23,24]. A volume of 5 μl of each overnight culture was spotted onto the surface of an MBS agar plate and allowed to soak in. Bacterial cultures were pre-grown for 48 hours and then flooded with 1 ml of a 1% (w/v) solution of each PAH dissolved in acetone. After evaporating the acetone, the plates were incubated at 30°C for up to 2 weeks and inspected daily for the presence of a clear zone surrounding the area of growth (scored positive).

### Heavy metal and metalloid ion resistance

Analytical grade heavy metal salts (3CdSO_4_ × 8H_2_O, CoSO_4_ × 7H_2_O, CuSO_4_, HgCl_2_, K_2_Cr_2_O_7_, NaAsO_2_, Na_2_HAsO_4_ × 7H_2_O, NiCl_2_ × 6H_2_O, ZnSO_4_ × 7H_2_O) were used to prepare 0.01 M, 0.1 M and 1 M stock solutions in water. Each solution was filter-sterilized and added to LB medium to produce a range of final concentrations (33 separate dilutions) of between 0.01 mM and 100 mM of the metal ion. Minimum inhibitory concentrations (MICs) for all analyzed strains were defined on titration plates using a broth dilution method in which changes in the optical density of cultures were measured in comparison with non-inoculated controls. Each microplate was monitored for growth using an automated microplate reader at 24-hour intervals for three days.

The heavy metal resistance phenotype was assessed from the ability to grow in the presence of (i) 10 mM As (V), (ii) 1 mM each of As(III), Cd, Co, Cu, Ni, Zn and Cr, and (iii) 0.1 mM Hg [25,26].

### Beta-lactams resistance

The MICs of antibiotics representing three classes of beta-lactams were determined by Epsilometer tests (E-tests, OXOID) using a gradient of the appropriate antibiotic: ampicillin (a penicillin), ceftazidime (a cefalosporin) and meropenem (a carbapenem). Each E-test strip was placed on lawns of the bacteria on agar plates and the pattern of growth was recorded after 48 hours incubation at 30°C or 37°C. The lowest concentration of the antibiotic that prevented growth was considered the MIC.

### Siderophore detection

The ability to produce siderophores was examined using the modified chrome azurol S (CAS) agar plate method [27]. Plates were incubated at 30°C for 72 hours in the dark and the formation of halos around colonies was recorded.

### Plasmid DNA isolation, genetic manipulations, PCR conditions and introduction of plasmid DNA into bacterial cells

The isolation of plasmids, Southern hybridization analysis and common DNA manipulation methods were performed as described by Sambrook and Russell [21]. PCR was performed in a Mastercycler (Eppendorf) using HiFi polymerase (Qiagen; with supplied buffer), dNTP mixture and total DNA of *Halomonas* sp. ZM3 with appropriate primer pairs: (i) LISPHSP1 (5'-GATAAGCGCCAGGCACCACA-3') and RISPHSP1 (5'-TCGGCGAGCTTCCTCAGAAC-3') – specific to IS*Hsp1*; (ii) LISPHSP2 (5'-TGTCCTCCGCCTATCACCAC-3') and RISPHSP2 (5'-ACGGCAGCCATGCGTACTTC-3') – specific to IS*Hsp2*; (iii) LCZCZM3 (5'-GATGCGCTCACCTCTGTATT-3') and RCZCZM3 (5'-CACAAGTGATGCGTTATCCG-3') – specific to the cobalt, zinc, cadmium (CZC) resistance module (*orf11*-*12*) of plasmid pZM3H1; and (iv) LMERZM3 (5'-GCGGAACCTGCGTCAACATT-3') and RMERZM3 (5'-GGCCATCACAGCAGTCTGAA-3') – specific to the mercury (MER) resistance module (*merA*, *orf19*) of pZM3H1. The *sacB-*specific primers used to identify the target site of transposition, were previously described by Szuplewska and Bartosik [20]. A colony PCR method for the amplification of 16S rRNA genes [28], used primers 27f and 1492r [29]. The transformation of *E. coli* strains was performed according to the method of Kushner [30]. Triparental mating was performed as described previously [31].

### Identification and analysis of a pool of TEs

Trap plasmid pMAT1 [20], containing *sacB* of *Bacillus subtilis*, was introduced into *Halomonas* sp. ZM3R. Overnight cultures of the kanamycin and rifampin resistant transconjugants were spread on plates of solidified LB medium supplemented with sucrose. The *sacB* gene encodes levansucrase, an enzyme whose activity (in the presence of sucrose) leads to accumulation of toxic compounds in the bacterial cell [32]. Therefore, cultivation of cells carrying the functional *sacB* gene in medium containing sucrose results in cell lysis. This allows direct selection of *sacB* mutants (Suc^r^) (e.g. carrying inserted TEs), whose growth is not affected under these conditions. The plasmids of 100 Suc^r^ clones were analyzed for the presence of inserted TEs.

### DNA sequencing

The complete nucleotide sequence of plasmid pZM3H1 was determined by the DNA Sequencing and Oligonucleotide Synthesis Laboratory (oligo.pl) at the Institute of Biochemistry and Biophysics, Polish Academy of Sciences. High-throughput sequencing of the MID-tagged shotgun plasmid-library was performed using an FLX Titanium Genome Sequencer (Roche/454 Life Sciences). Newbler *de novo* assembler software (Roche) was used for the sequence assembly. Final gap closure and sequence polishing were performed by capillary sequencing of PCR products using an ABI3730xl DNA Analyzer (Applied Biosystems).

Nucleotide sequences of the insertion sequences were obtained using the primer walking approach with a dye terminator sequencing kit and an automated sequencer (ABI 377 Perkin Elmer; oligo.pl).

### Bioinformatics

Plasmid nucleotide sequences were analyzed using Clone Manager (Sci-Ed8) and Artemis software [33]. Similarity searches were performed using the BLAST programs [34] provided by the National Center for Biotechnology Information (NCBI) (http://blast.ncbi.nlm.nih.gov/Blast.cgi) and the PRIAM tool [35]. Comparison searches of insertion sequences were performed with ISfinder [36]. Helix-turn-helix motifs were predicted using the HELIX-TURN-HELIX MOTIF PREDICTION program [37]. Phylogenetic analyses were performed using the Phylogeny Inference Package – PHYLIP v3.69 [38], applying the neighbor-joining (NJ) algorithm with Kimura corrected distances and 1000 bootstrap replicates. DNA sequence alignments obtained with ClustalW [39] were manually refined using the T-Coffee Multiple Sequence Alignment program [40]. Highly variable portions of the alignments were eliminated by the use of G-blocks [41]. The tree was rendered with TreeView version 1.6.6. [42].

### Nucleotide sequence accession numbers

The 16S rDNA sequence of *Halomonas* sp. ZM3 has been deposited in the NCBI database with the accession number [GenBank:JX569337]. The nucleotide sequences of plasmid pZM3H1 and insertion sequences IS*Hsp1* and IS*Hsp2* have been annotated and deposited with the accession numbers [GenBank:JX569338], [GenBank:JX569339] and [GenBank:JX569340], respectively.

## Results

### Physiological characterization of the strain ZM3

A comparative analysis of the partial 16S rDNA sequence (1409 bp) of strain ZM3 revealed a high level of similarity to the corresponding sequences of several environmental isolates of *Halomonas* spp. (98.87%) and *Halomonas variabilis* DSM 3051^T^ (97.89%) isolated from the Great Salt Lake (Utah, USA) [43]. Based on this sequence homology, the strain ZM3 was classified in the genus *Halomonas*.

To identify specific features of *Halomonas* sp. ZM3 that have enabled its adaptation to the extreme environment of Zelazny Most, a complex physiological characterization of the strain was performed, including analyses of (i) temperature, pH and salinity tolerance, (ii) siderophore production, (iii) resistance to heavy metal ions, and (iv) PAH utilization ability. The obtained results revealed that strain ZM3 can grow in LB medium at temperatures ranging from 15 to 37°C (typical for mesophilic bacteria), but within a relatively narrow pH range of between 6 and 8 (typical for neutrophilic bacteria; [44]). Moreover, it can tolerate high salinity (up to 12% NaCl in the growth medium) and the presence of high concentrations of inorganic arsenic species (MICs for As(III) and As(V) of 9 mM and 700 mM, respectively). A low level of resistance to copper, mercury and nickel was also observed (Table 1). Analysis of the pattern of PAH utilization (five tested compounds – anthracene, phenanthrene, fluoranthene, fluorene and pyrene) revealed that strain ZM3 uses phenanthrene as the sole source of carbon. Application of the universal chrome azurol S (CAS) agar plate assay demonstrated that the ZM3 strain produces high levels of iron-chelating siderophores (data not shown).

**Table 1:** **Heavy metal resistance of *Halomonas *sp. ZM3**

**Heavy metal resistance**	
Metal	MIC
	(mM)
As (III)	**9**
As (V)	**700**
Cd (II)	0.2
Co (II)	0.7
Cr (VI)	1
Cu (II)	**3**
Hg (II)	**0.1**
Ni (II)	**2**
Zn (II)	0.7

MICs considered to represent the resistance phenotype shown in bold.

The results of these physiological tests revealed that *Halomonas* sp. ZM3 is well adapted to inhabit the Zelazny Most mineral waste reservoir. Since many features of adaptive value are frequently determined by mobile genetic elements (e.g. widely disseminated plasmids and transposons), we analyzed the extrachromosomal DNA of this strain.

### General features of plasmid pZM3H1

**Figure 1:**
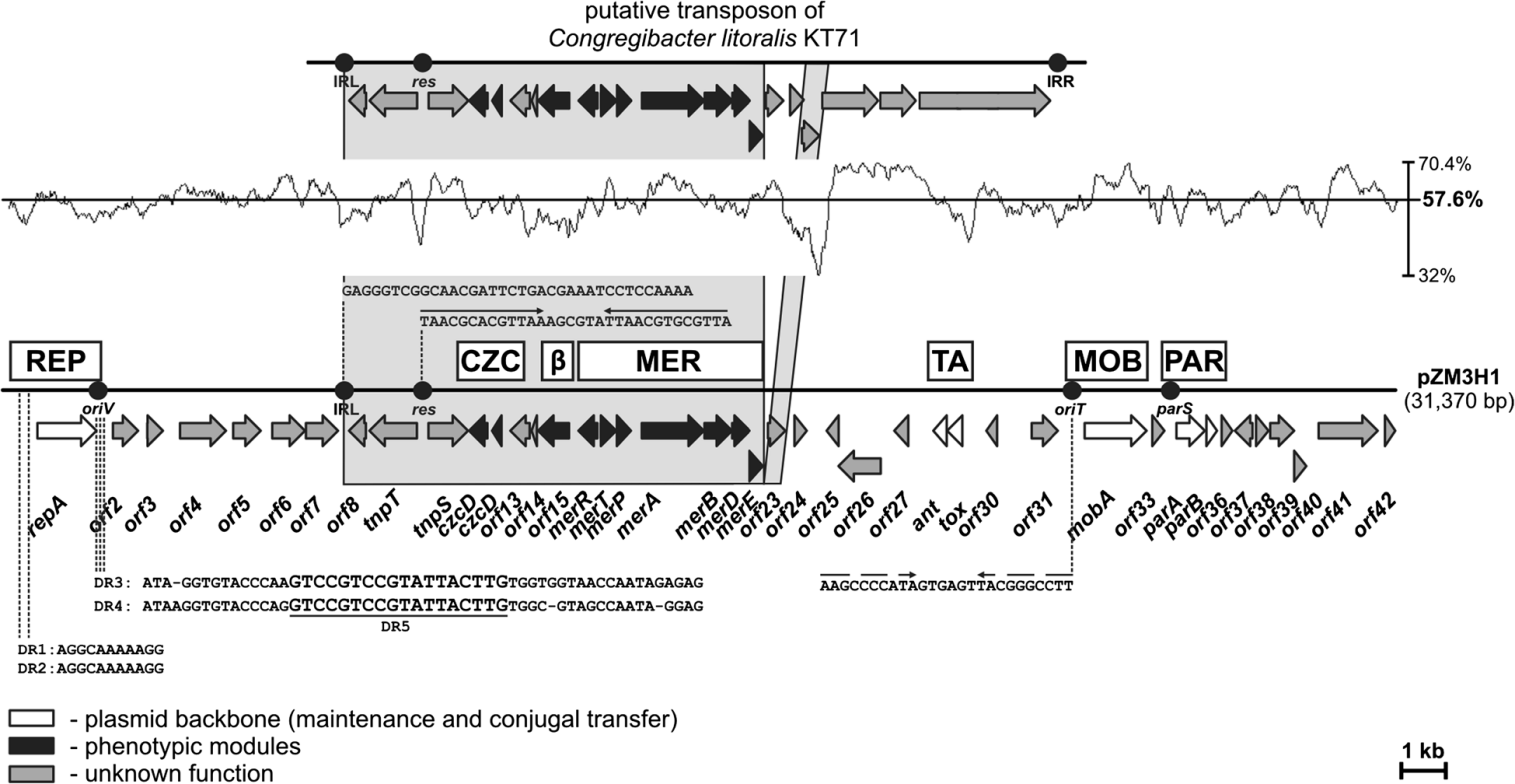
**Linear map showing the genetic structure of circular plasmid pZM3H1.** The predicted genetic modules are indicated by white rectangles: REP – replication system, CZC – cobalt, zinc and cadmium resistance module, β – putative beta-lactamase, MER – mercury resistance module, TA – toxin-antitoxin system, MOB – system for mobilization for conjugal transfer, PAR – partitioning system. Arrows indicate the transcriptional orientation of the genes. The plot shows the G+C content of the pZM3H1 sequence (mean value 57.6 mol%). The gray-shaded area connects genes of plasmid pZM3H1 and *C. litoralis* KT71 that encode orthologous proteins. Sequences and structures of *cis*-acting elements responsible for plasmid replication (*oriV*), maintenance (*parS*), mobilization (*oriT*), as well as elements of a putative transposon (IRL and *res*) are shown. DR – direct repeats within the REP module.

*Halomonas* sp. ZM3 carries only one extrachromosomal replicon, designated pZM3H1. DNA sequencing demonstrated that pZM3H1 is a circular plasmid (31,370 bp) with a mean G+C content (determined from its nucleotide sequence) of 57.6% (Figure 1). More detailed *in silico* analyses revealed that pZM3H1 carries 42 putative open reading frames (ORFs) (73.6% of the sequence). Three of them (*orf5*, *orf27, orf39*) have no homologs in public databases, while 15 have homologs of unknown function. The functions of the remaining ORFs were predicted from their similarities to known protein coding sequences. Features of these ORFs, including their position, transcriptional orientation, the size of the encoded proteins, and their closest known homologs, are summarized in Additional file 1: Table S1).

Further analysis of pZM3H1 revealed its modular structure. Within the plasmid genome it was possible to distinguish putative genetic modules responsible for (i) plasmid maintenance – replication (REP) and stabilization, (ii) mobilization for conjugal transfer (MOB), (iii) resistance to heavy metals, and (iv) other accessory genetic information (Figure 1).

### Characterization of the conserved backbone of plasmid pZM3H1

The backbone of pZM3H1 is composed of (i) a REP module (*orf1*), (ii) a MOB module (*orf32*) and two types of stabilization module, namely (iii) PAR (*orf34*-*orf35*), encoding a partitioning system responsible for the correct distribution of plasmid molecules into daughter cells upon cell division, and (iv) TA (*orf28*-*orf29*), encoding a toxin and antitoxin involved in postsegregational elimination of plasmid-less cells (Figure 1).

The REP module of pZM3H1 carries a single ORF (*orf1*) encoding a predicted protein with similarities to the RepA replication initiation proteins of several bacterial plasmids, including two well characterized members of the IncU incompatibility group: plasmid RA3 of *Aeromonas hydrophila*[45] and Rms149 of *Pseudomonas aeruginosa*[46]. The predicted RepA of pZM3H1 (as well as other related replication proteins) contains a putative helix-turn-helix (HTH) motif (FSYRKIATAMETSVSQVQRMLT; residues 420–441) located within the *C*-terminal part of the protein.

The putative *repA* gene (*orf1*) is bordered on both sides by stretches of A+T-rich sequence (AT content of approx. 47.5%). Analysis of the upstream region revealed the presence of two identical direct repeat sequences (DR1, DR2; 5^′^-AGGCAAAAAGG-3′) separated by a spacer of 327 bp. The downstream region contains two long (52 and 51 bp), nearly identical (3 differences) direct repeats (DR3, DR4) separated by an 87-bp spacer (Figure 1). It is noteworthy that the four 5^′^-terminal residues of DR3 are located within the RepA coding sequence. Moreover, a shorter sequence was identified 91 bp upstream of DR4 (DR5; 5^′^-GTCCGTCCGTATTACTTG-3^′^), that perfectly matches the core region of the DR3 and DR4 repeats (Figure 1).

Such repeated sequences, placed downstream and upstream of the *repA* gene, were also identified within the REP region of the related plasmid RA3. It was demonstrated that the downstream repeats are crucial for the initiation of RA3 replication [45]. Based on the overall similarities of the REP regions, we assume that the origin of replication of pZM3H1 (*oriV*) is placed analogously to that of RA3, and contains the DR3, DR4 and DR5 repeats (Figure 1).

The putative PAR module of pZM3H1 is composed of two non-overlapping ORFs (*orf34* and *orf35*; 31-bp spacer) and a centromere-like site. The *orf34* encodes a putative 214-aa protein, showing significant similarity to ATPases involved in chromosome partitioning, assigned to COG1192 (cluster of orthologous group). This similarity includes the sequence KGGVGKS (residues 11–17), which matches the highly conserved canonical deviant Walker A motif KGG(T/N/V)GKT of ParA-type proteins [47]. This predicted ParA also contains an *N*-terminally located putative HTH motif (YIIGVVSQKGGVGKSTISRAVAT; residues 3–24). The *orf35* encodes an 80-aa polypeptide with sequence similarity to several hypothetical proteins, whose genes are usually located downstream from predicted *parA* genes (i.e. *orf34* homologs). This strongly suggests that *orf35* encodes a ParB-type protein: another important component of plasmid partitioning systems. Careful inspection of the nucleotide sequence revealed the presence of several 7-bp imperfect inverted repeats, located close to the promoter region of the predicted *par* operon, which may constitute a plasmid centromere-like site (*parS*) (Figure 1).

TA stabilization modules usually encode two components: a toxin which recognizes a specific cellular target and an antitoxin, which counteracts the toxin. The predicted TA module of pZM3H1 fits with this scheme, since it is composed of two short non-overlapping ORFs (*orf29* and *orf28*) separated by a 9-bp spacer. One of the ORFs (*orf29*) encodes a putative protein with significant sequence homology to a large family of proteins assigned to COG4679 (DUF891). These proteins, referred to as phage-related (some are encoded by bacteriophages, e.g. gp49 of phage N15), were shown to be the toxic components (RelE/ParE toxin family) of a number of TA systems [48]. The downstream gene (*orf28*) encodes a putative protein with substantial similarity to antitoxins classified to COG5606 and COG1396. The predicted antitoxin contains a HTH domain typical for members of the Xre/Cro protein family. Pairs of genes homologous to *orf29*-*orf28* are conserved and widely distributed in bacterial genomes, including the *tad-ata* locus of plasmid pAMI2 of *Paracoccus aminophilus* JCM 7686 – the archetype of this group of TA systems [48].

The next component of the pZM3H1 backbone, the MOB module, encodes a single mobilization protein (Orf32/MobA) sharing a low, but significant level of amino acid (aa) sequence homology with the Mob relaxases of pOCEGK02 from *Oceanimonas* sp. GK1 [GenBank: NC_016747] and broad-host-range plasmid pBBR1 of *Bordetella bronchiseptica* S87 [GenBank:X66730] (33% and 31% similarity, respectively). Detailed comparative sequence analysis of the potential Orf32/MobA relaxase revealed the presence of several conserved motifs, which permits classification of the protein into the MOB_V2_ group within the MOB_V_ family [49]. Upstream of the putative *mobA* (*orf39*) gene, an imperfect (2 mismatches) 10-bp inverted repeat sequence was identified (5^′^-AAGCCCCATAGTGAGTTACGGGCCTT-3^′^; nt position 24,073-24,098), whose location and structure is typical for the *origin* of conjugal transfer (*oriT*) of MOB systems encoding MOB_V_ type relaxases (e.g. [50]).

### Analysis of the host range of pZM3H1

To analyze the host range of the *Halomonas* sp. ZM3 plasmid, a mobilizable shuttle replicon pABW-ZM3H1 was constructed, containing the REP module of pZM3H1 and an *E. coli*-specific pMB1 (ColE1-type) replication system (see Methods for details). The obtained plasmid was introduced *via* conjugation into strains representing three classes of *Proteobacteria*: (i) *Alpha*- (*A. tumefaciens* LBA288 and *P. versutus* UW225), (ii) *Beta*- (*Alcaligenes* sp. LM16R), and (iii) *Gammaproteobacteria* (*Pseudomonas* spp. – strains LM5R, LM6R, LM7R, LM8R, LM11R, LM12R, LM13R, LM14R, LM15R). The plasmid was also introduced by transformation into *E. coli* BR825 (*Gammaproteobacteria*). Since the *E. coli*-specific system is not functional in any of the strains listed above (*E. coli* BR825 carries a mutation within the DNA polymerase I gene that prevents pMB1 replication), the functions required for replication of the plasmid in the tested hosts must be provided by the REP module of pZM3H1. This analysis demonstrated that pABW-ZM3H1 could replicate exclusively in two *Pseudomonas* strains (LM7R and LM12R), which indicates a relatively narrow host range.

### Characterization of the resistance modules

Comparative sequence analysis revealed that a large DNA segment of pZM3H1 (10.1 kb; coordinates 7594–17,726) is highly conserved (95% nucleotide sequence identity) in the genome of *Congregibacter litoralis* KT71 (unfinished genome project [contig accession number – GenBank:NZ_AAOA01000001]). As shown in Figure 1, the homologous *C. litoralis* region differs slightly, since it contains two additional ORFs (encoding a putative DoxD-like membrane protein and a truncated transposase) that are absent in pZM3H1 (Figure 1). Further *in silico* sequence analysis revealed that this region of the *C. litoralis* genome represents part of a putative Tn*3* family transposon (coordinates 155,646-171,707 [GenBank:NZ_AAOA01000001]) (Figure 1), related to Tn*4651* identified in plasmid pWW0 of *Pseudomonas putida*[51]. Plasmid pZM3H1 carries a large portion of this *C. litoralis* transposon (17 ORFs; *orf8-orf23*), although it lacks the 5.3 -kbterminal region of the element, which contains three genes coding for a putative NADP-specific glutamate dehydrogenase, a conserved membrane protein and a transposase (Figure 1). This truncated transposon contains (i-ii) two heavy metal resistance cassettes – a Co/Zn/Cd efflux system (*orf11*, *orf12*) and mercury resistance determinants (*orf16*-*orf22*), (iii) an ORF encoding a protein of the metallo-beta-lactamase family (*orf15*), (iv) a site-specific resolution system (composed of two genes *tnpS* and *tnpT*, and a putative resolution site with a hairpin structure) homologous to the MRS system of Tn*4651*[51], as well as (v) four ORFs encoding hypothetical proteins with unknown functions (*orf8*, *orf13*, *orf14* and *orf23*) (Figure 1).

The putative efflux system (CZC module; *orf11, orf12*) encodes a predicted CzcD metal transport membrane protein (a member of the cation diffusion facilitator protein family), which mediates cobalt (Co^2+^), zinc (Zn^2+^) and cadmium (Cd^2+^) resistance (as shown in *Cupriavidus metallidurans* CH34 [52]). The mercury resistance module (MER) contains 7 ORFs (*orf16*-*orf22*) with significant levels of homology to the *merRTPABDE* genes, responsible for enzymatic detoxification of Hg^2+^ ions to the less toxic form Hg^0^[53]. The key enzymes in this mercury resistance system are (i) organomercurial lyase (MerB) – effectively performs hydrolysis of stable mercury-carbon bonds, and (ii) mercuric reductase (MerA) – reduces Hg^2+^ to Hg^0^ (metallic mercury) in a process that involves hydride transfer from the electron carrier NADPH to flavin. Three other important components are (i) two transcriptional regulatory proteins (MerR and MerD), (ii) two mercury ion transport proteins (MerT and MerP), and (iii) an accessory membrane protein (MerE) [53] (Figure 1 and Additional file 1: Table S1).

**Figure 2:**
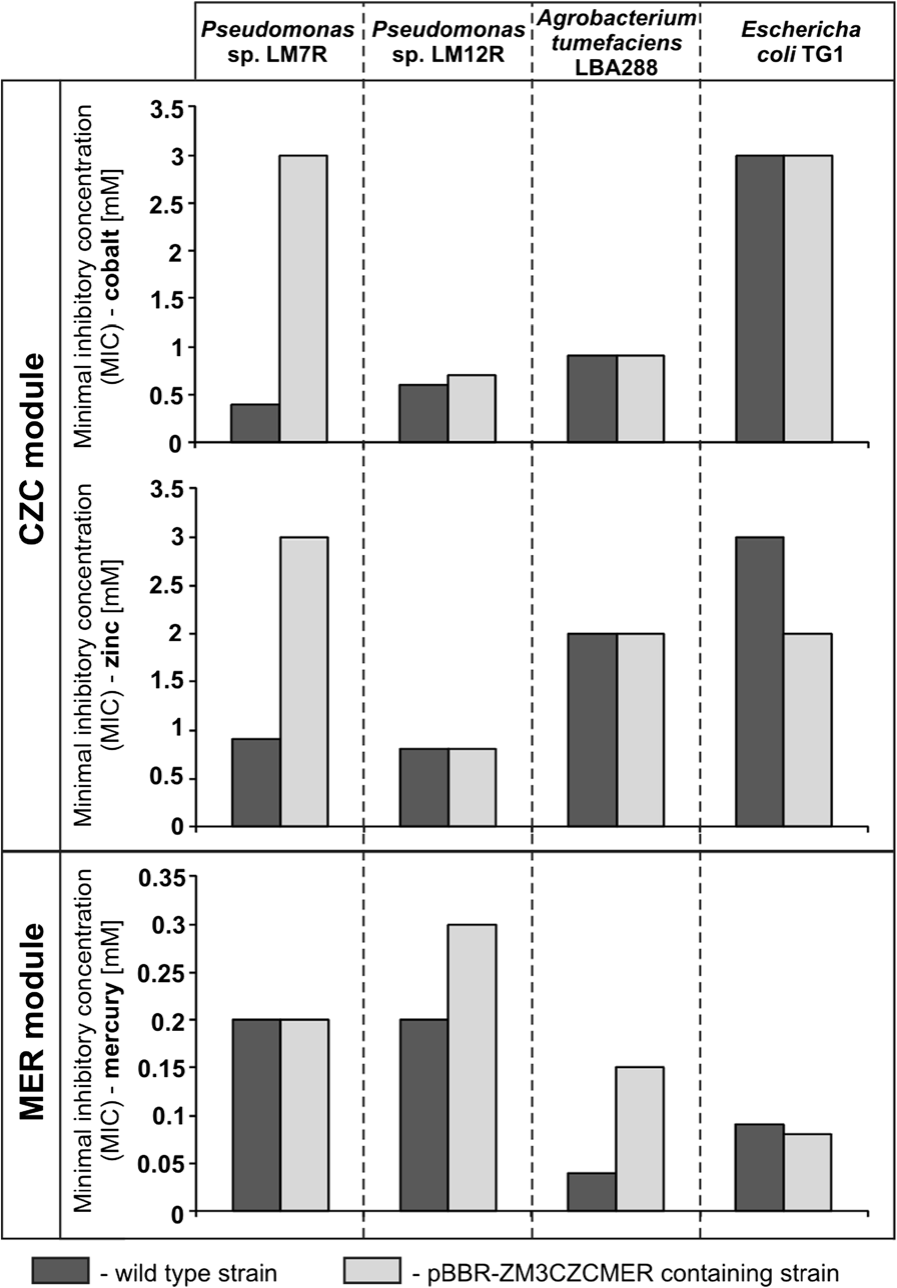
**Resistance phenotypes determined by the CZC and MER modules.** MICs of cobalt, zinc and mercury ions for wild-type strains (dark gray) and strains carrying pBBR-ZM3CZCMER (the plasmid contains CZC and MER resistance modules) (light gray) of *Pseudomonas* sp. LM7R, *Pseudomonas* sp. LM12R, *A. tumefaciens* LBA288 and *E. coli* TG1.

To investigate whether the analyzed resistance cassettes are functional, plasmid pBBR-ZM3CZCMER was constructed by inserting the *orf11-orf23* gene cluster of pZM3H1 (contains the CZC and MER modules) into broad-host-range (BHR) mobilizable vector pBBR-MCS2 (see Methods for details). Since we were unable to remove (by incompatibility) plasmid pZM3H1 from its natural host (*Halomonas* sp. ZM3), the obtained plasmid pBBR-ZM3CZCMER was introduced (by conjugation or transformation) into *Pseudomonas* spp. LM7R and LM12R (pZM3H1 was shown to replicate in both strains) and *E. coli* TG1 (three members of *Gammaproteobacteria*), as well as *A. tumefaciens* LBA288 (*Alphaproteobacteria*). The resistance phenotypes of the obtained transconjugants and the wild-type strains were then tested by determination of the minimum inhibitory concentrations (MICs) of Cd, Co, Zn and Hg salts in liquid culture (Figure 2).

This analysis revealed that introduction of pBBR-ZM3CZCMER into strain LM7R resulted in a significant increase in the MICs of cobalt (6-fold) and zinc (3-fold), which indicates resistance. In contrast, the level of tolerance to mercury was not changed (Figure 2). Different results were obtained with the transconjugants of strains LM12R and LBA288, which exhibited resistance to mercury (MIC increases of 1.5- and 3-fold, respectively), but not to cobalt or zinc. Interestingly, none of the tested strains was resistant to cadmium. Introduction of the plasmid pBBR-ZM3CZCMER into *E. coli* TG1 did not result in cobalt or mercury resistance; however, an unexpected increase in sensitivity to zinc was observed (Figure 2).

Besides the CZC and MER modules, plasmid pBBR-ZM3CZCMER also carries *orf15* encoding a protein related to metallo-beta-lactamases, many of which confer resistance to beta-lactam antibiotics, e.g. [54]. Therefore, we tested whether the pBBR-ZM3CZCMER-containing strains (LM7R, LM12R, LBA288, TG1) acquired resistance to antibiotics representing three classes of beta-lactams: (i) ampicillin (penicillins), (ii) ceftazidime (cefalosporins) and (iii) meropenem (carbapenems). The MICs, determined by Epsilometer tests, revealed no resistance phenotype, indicating that Orf15 protein does not exhibit beta-lactamase activity in these strains.

### Identification and characterization of transposable elements (TEs)

For the identification of functional TEs of *Halomonas* sp. ZM3 we employed the mobilizable BHR trap plasmid pMAT1, carrying the *sacB* cassette, which enables positive selection of transposition events [20]. A pool of putative transposition mutants was collected and analyzed as described in Methods. From this set of mutants, two classes of pMAT1 derivatives were identified, containing inserted elements of respective sizes 1 kb and 1.5 kb, which is typical for the majority of insertion sequences (ISs). DNA sequencing and comparison of the obtained nucleotide sequences (NCBI and ISfinder databases) revealed that the identified elements were novel insertion sequences, designated IS*Hsp1* and IS*Hsp2*.

**Figure 3:**
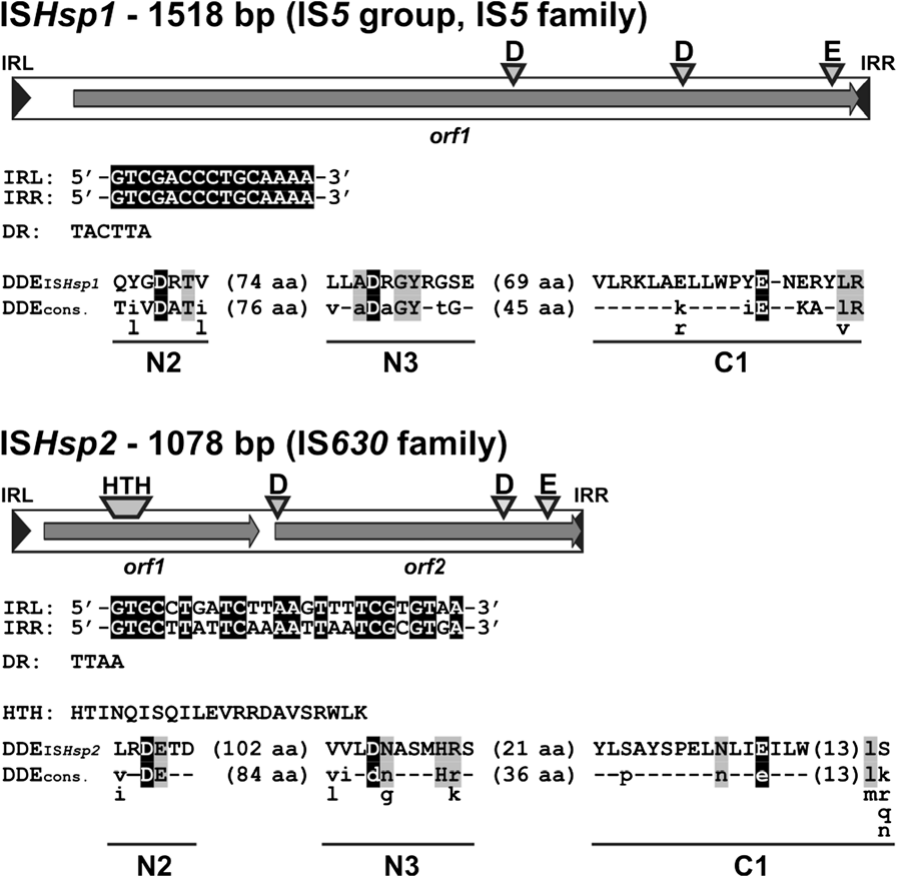
**Genetic organization of the insertion sequences IS*Hsp1 *and IS*Hsp2*.** Inverted repeats (IRL – left IR; IRR – right IR) flanking ISs are marked by black arrowheads. Predicted coding regions are represented by gray arrows indicating the direction of transcription. The location of the DNA-binding domain (HTH) and the DDE motifs are marked. Alignments of the inverted terminal repeats and the sequences of the duplicated direct repeats (DR) are presented beneath each insertion sequence diagram. Identical nucleotides within the IRL and IRR of each IS are indicated by white text against a black background. The amino acid sequences of the predicted N2, N3, and C1 regions, and DDE motifs of the putative transposases encoded by IS*Hsp1* and IS*Hsp2* are compared with appropriate family- and group-specific consensus sequences. In the consensus sequences, uppercase letters indicate conservation within the family or group, while lowercase letters denote predominant amino acids, and dashes mark the non-conserved residues. Residues forming the DDE motif are indicated by white text on a black background. The residues conserved in the domains of the analyzed transposases and the consensus sequences are presented against a gray background. The numbers in parentheses show the distances (in amino acids) between the conserved domains.

IS*Hsp1* carries identical terminal inverted repeat sequences (IRs) of 15 bp at both ends (Figure 3). Transposition of the element into the *sacB* cassette of pMAT1 resulted in duplication of a short (6 bp) target sequence (5^′^-TACTTA-3^′^) to form direct repeats (DRs) (Figure 3). Within the 1518-bp-long sequence of IS*Hsp1* (G+C content – 56.7%) only one ORF was identified (nt position 113–1495), encoding a putative protein (460 aa; 52.7 kDa) with 93% sequence identity to the transposase of IS*Maq6* of *Marinobacter aquaeolei* VT8 [GenBank:ABM18542]. Both elements (IS*Hsp1* and IS*Maq6*) also show high overall similarity (89%) of their nucleotide sequences.

The predicted transposase of IS*Hsp1* contains N2, N3 and C1 regions, including three acidic residues (DDE motif), that are highly conserved in the catalytic domains of transposases of bacterial TEs and retrovirus integrases [55]. As shown in Figure 3, the sequence of this motif is in relatively good agreement with the DDE consensus for transposases of the IS*5* group of the IS*5* family; however, the distance between the N3 and C1 regions (69 aa) is significantly longer than that of the consensus sequence (45 aa).

The other captured element, IS*Hsp2* (1078 bp; G+C content – 53.7%), contains non-identical terminal IRs of 26 bp (10 mismatches) and two non-overlapping ORFs (*orf1* and *orf2*), encoding putative proteins of 132 aa (15.2 kDa) and 192 aa (22.2 kDa), respectively (Figure 3). Within *orf1* (nt position 446), a putative −1 frameshift motif was identified (5^′^-GAAAAAAAAA-3^′^) in the loop of a predicted mRNA stem-loop structure. This motif most probably promotes a programmed translational frameshift, which leads to the formation of a functional fusion (Orf1+Orf2) transposase (as shown e.g. for IS*1* and IS*3* family members [56,57]). The putative proteins encoded by the individual ORFs of IS*Hsp2* carry a potential HTH DNA-binding motif (Orf1) and a complete DDE motif (Orf2) – typical for the IS*630* family. Both motifs are also present within the predicted trans-frame transposase (337 aa; 40 kDa) generated by translational slippage. The fusion transposase shows the highest level of aa sequence similarity (44%) with the fusion transposase of IS*Vsa8* – an element present in 27 copies in chromosomes I and II of *Aliivibrio salmonicida* LFI1238 [58].

Based on the results of the comparative analysis, IS*Hsp1* and IS*Hsp2* have been classified as novel members of the IS*5* (IS*5* group) and IS*630* families, respectively.

### Copy number of selected genes of the identified MGEs

To determine the copy number of the identified ISs, as well as the CZC and MER modules of pZM3H1 in the *Halomonas* sp. ZM3 genome, DNA hybridization analysis was performed. PCR-amplified digoxygenin-labeled internal fragments of the *merA* gene (*orf19*), *czcD* gene (*orf11-12*), IS*Hsp1* and IS*Hsp2* (primers used are given in Methods) were used to probe Southern blots of total *Halomonas* sp. ZM3 DNA digested with restriction enzymes (selected so that the number of DNA fragments hybridizing with the probes was equivalent to the minimum number of copies of a given gene/element). Using this method, single copies of the CZC and MER modules were identified in the ZM3 genome (within plasmid pZM3H1), while four and two copies of the insertion sequences IS*Hsp1* and IS*Hsp2* were detected, respectively (data not shown).

## Discussion

Groundwater from the Lubin-Glogow mines (Copper Mine District, Poland), that has various uses including the flotation of sulfides during ore processing, contains sodium and chlorine ions at high concentrations, which results in elevated salinity of the deposits in the Zelazny Most waste reservoir. These conditions favor the expansion of halophilic microorganisms, one of which (*Halomonas* sp. ZM3) was analyzed in this study. Strain ZM3 is well adapted to the harsh environment of Zelazny Most, since it is hyper-resistant to inorganic arsenic species As(III) and As(V), and shows elevated resistance to highly toxic ions of copper, mercury and nickel. Moreover, it is able to utilize phenanthrene (composed of three fused benzene rings), which makes it a good candidate for bioremediation of PAH-contaminated hypersaline environments. The strain ZM3 is also an efficient siderophore producer. Such metal chelating compounds have been shown to complex iron and other metals, and also mobilize chemical elements from minerals (e.g. hausmannite [59]), and so may play a significant role in the redistribution of elements in the Zelazny Most environment.

The present study was focused on mobile genetic elements (plasmid and TEs) of *Halomonas* sp. ZM3. Our analysis revealed that the ZM3 strain carries only one extrachromosomal replicon (plasmid pZM3H1; 31,370 bp), whose replication system shows similarity to the REP modules of several plasmids classified within the IncU incompatibility group. Although this group contains several broad-host-range conjugative plasmids responsible for the dissemination of antibiotic resistance determinants (e.g. [60]), there is a dearth of knowledge about IncU replicons. To better define this group, we performed comprehensive BLAST searches of the NCBI database, which identified 27 replicons encoding homologous Rep proteins (E-value < 1 × 10^-20^) with amino acid sequence similarity levels of ≥27%.

**Figure 4:**
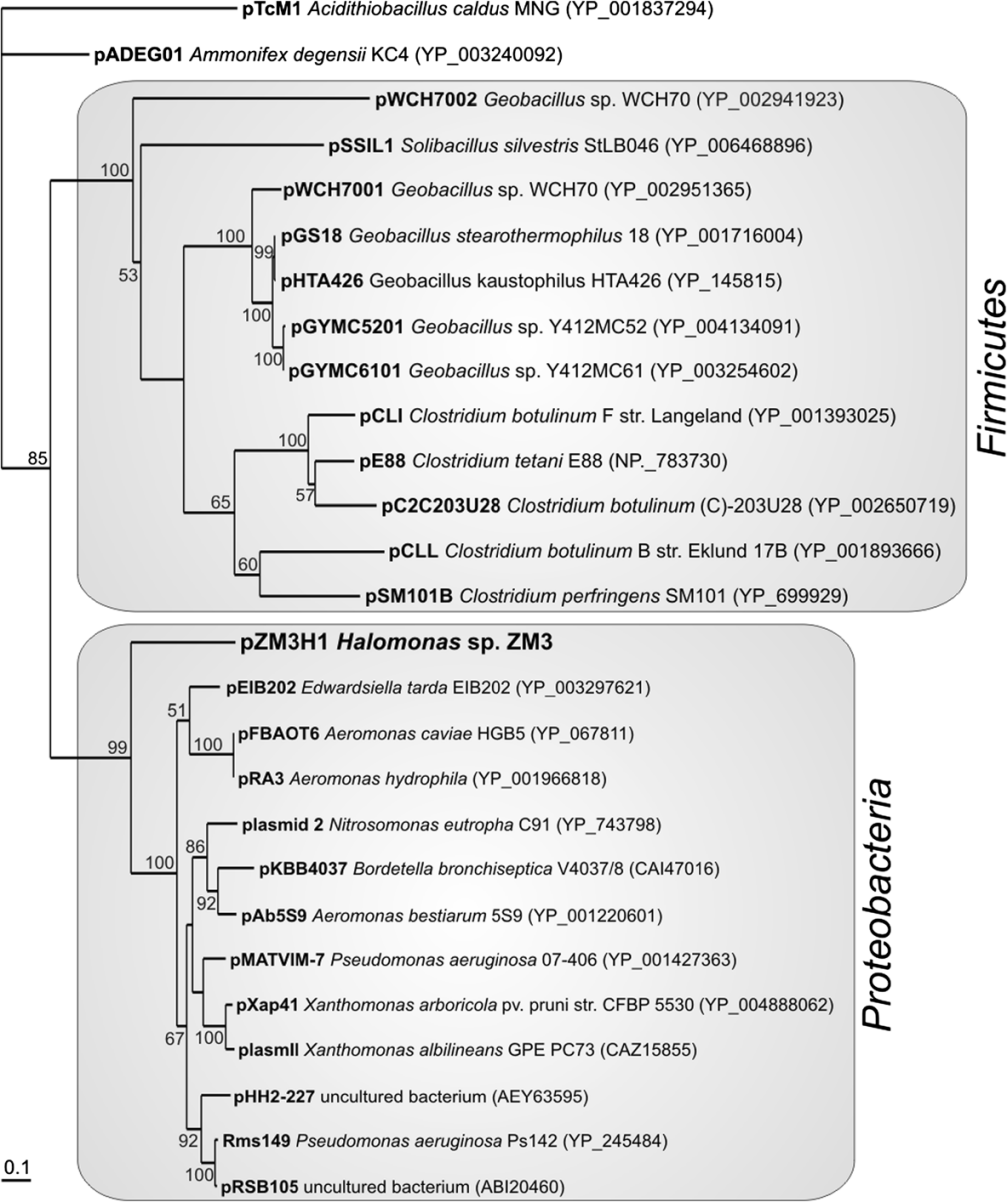
**Phylogenetic tree of the replication initiation protein (Rep) of IncU-family plasmids.** The analysis was based on 27 sequences (from fully sequenced plasmids) and 217 amino acid positions. The unrooted tree was constructed using the neighbor-joining algorithm with Kimura corrected distances, and statistical support for the internal nodes was determined by 1000 bootstrap replicates. Values of >50% are shown. Accession numbers of the protein sequences used for the phylogenetic analysis are given in parentheses.

Phylogenetic analysis of the IncU plasmids (performed on the basis of the Rep protein sequences) revealed the presence of two subgroups, comprised of 12 and 13 replicons, which clearly correspond to the Gram-negative (*Proteobacteria*) and Gram-positive (*Firmicutes*) hosts, respectively. As shown in Figure 4, the phylogenetic distance of the pZM3H1 Rep reflects its weak relationship with Rep proteins of Gram-negative bacteria. This suggests that the replication system of pZM3H1 may be considered as an archetype of a novel subgroup of IncU-like replicons (Figure 4).

The divergence of the REP module may be reflected by the relatively narrow host range (NHR) of pZM3H1. Besides the native strain ZM3, this plasmid was shown to replicate in only two (of nine tested) strains of *Pseudomonas* (isolated from the Lubin copper mine). Many of the analyzed strains lack their own plasmids, so the failure to obtain transconjugants did not result from incompatibility between the incoming and residing replicons. Therefore, it may be hypothesized that the initiation of pZM3H1 replication requires specific cellular factors present only in some strains or species of the genus *Pseudomonas* or *Halomonas*.

Plasmid pZM3H1 contains a predicted MOB module, which suggests that it may be mobilized for conjugal transfer. It has recently been demonstrated that the host range of MOB systems can be wider than the replication systems of the plasmids they carry. Thus, NHR mobilizable plasmids may be considered as efficient carrier molecules, which act as natural suicide vectors promoting the spread of diverse genetic information (e.g. resistance transposons) among evolutionarily-distinct bacterial species [61]. Plasmid pZM3H1, despite its narrow host range, may therefore play an important role in horizontal dissemination of genetic modules conferring heavy metal resistance phenotypes.

The resistance cassette of pZM3H1, composed of MER and CZC genetic modules, is part of a large truncated Tn*3* family transposon. It is well known that *mer* operons mediate detoxification of mercury compounds, while *czcD* genes mediate low level Zn^2+^, Co^2+^ and Cd^2+^ resistance (higher level resistance is usually determined by the *czcCBA* system) [62]. Both modules are widely disseminated in bacterial genomes and frequently occur on plasmids and transposons (e.g. [53,63]).

Unexpectedly, the introduction of these resistance modules into two *Pseudomonas* spp. strains (LM7R and LM12R – both able to maintain pZM3H1) produced completely different phenotypes. Strain LM7R (containing MER+CZC) gained resistance to zinc and cobalt, but not mercury, whereas LM12R acquired only mercury resistance (Figure 2). Moreover, neither of the strains was resistant to cadmium. This finding demonstrated that the phenotype determined by plasmid pZM3H1 is highly dependent on the host strain. The host specificity of resistance phenotypes generated by two related *czcD* modules of *Staphylococcus aureus* and *Thermus thermophilus* was also described by Nies [62]. The results revealed that the former is involved in zinc and cobalt resistance, while the latter mediates zinc and cadmium (but not cobalt) resistance.

In another strand of the present study, the trap plasmid pMAT1 was employed to identify functional transposable elements of *Halomonas* sp. ZM3. Using the *sacB* positive selection strategy, we were unable to “capture” any resistance transposons. The only identified elements were two insertion sequences: IS*Hsp1* (IS*5* group of IS*5* family) and IS*Hsp2* (IS*630* family). Both elements are present in more than one copy in the ZM3 genome, and so they may potentially form composite transposons.

IS*Hsp1* is most closely related to IS*Maq6* of *M. aquaeolei* VT8 (89% nucleotide sequence identity). Members of the genera *Marinobacter* and *Halomonas* are widely distributed in many environments. These bacteria are usually isolated from the same habitats, including oceans and seas, saline soils, marine snow, hot springs and volcanic basalts [64], which may favor horizontal gene transfer between them (several strains of *Marinobacter* spp. have been isolated from the Zelazy Most reservoir; unpublished results).

The second “captured” element, IS*Hsp2*, was classified within the IS*630*/Tc*1* superfamily, which is comprised of promiscuous TEs found in both prokaryotes and eukaryotes [65]. IS*Hsp2* carries two ORFs encoding the *N*- and *C*-terminal parts of the transposase, respectively. Therefore, generation of the complete functional enzyme requires ribosomal frame-shifting: a phenomenon that plays an important role in regulating the frequency of transposition of some ISs (e.g. [56,57]). The fusion transposase of IS*Hsp2* exhibits only a moderate level of amino acid sequence homology to transposases of the IS*630* family. Moreover, transposition of the IS generates 4-bp-long DRs (5^′^-TTAA-3^′^), while other related elements duplicate only the 5^′^-TA-3^′^ dinucleotide. These divergent features indicate that IS*Hsp2* represents a distinct member of the IS*630* family.

## Conclusions

Bacteria of the genus *Halomonas* are “opportunitrophic” microbes, since they are generalists that employ a strategy of acquiring and maintaining a broad and diverse metabolic potential in order to exploit changeable environmental resources [64]. Such strains may find application in biotechnology and bioremediation, so it is extremely important to characterize their metabolic potential, as well as their mobile genetic elements, which facilitate horizontal gene transfer and increase the fitness of their hosts in extreme environments.

The overall characterization of *Halomonas* sp. ZM3 has provided information concerning genus- (elevated salinity tolerance), as well as strain-specific physiological features (i.e. arsenic, copper, mercury and nickel resistance, and phenanthrene utilization ability), that enable the survival of ZM3 in the highly contaminated environment of Zelazny Most.

Special attention was given to plasmid pZM3H1, carrying heavy metal resistance determinants. This plasmid is unique among the elements identified in this genus (sequences from 8 *Halomonas* spp. genome projects), which suggests its relatively recent acquisition.

Characterization of the ZM3 plasmid as well as two novel transposable elements increase current knowledge concerning the diversity of mobile DNA of bacteria of the family *Halomonadaceae*. Moreover, the identified elements and their individual genetic modules may be used to construct specific tools for the genetic analysis of *Halomonas* spp.

## Abbreviations

BHR: Broad host range; COG: Cluster of orthologous group; CZC: Cobalt, Zinc and Cadmium Resistance Determinant; DR: Direct repeat sequences; DUF: Domain of unknown function; HTH: Helix turn helix; IS: Insertion sequence; LB: Lysogeny broth; MER: Mercury resistance determinant; MGE: Mobile genetic elements; MIC: Minimum inhibitory concentration; MOB: Mobilization for conjugal transfer; NADP: Nicotinamide adenine dinucleotide phosphate, ncbi, national center for biotechnology information; NHR: Narrow host range; ORF: Open reading frame; PAH: Polycyclic aromatic hydrocarbons; PAR: Partitioning; REP: Replication; TA: Toxin-Antitoxin; TE: Transposable element

## Competing interests

The authors declare that they have no competing interests.

## Authors’ contributions

LD and AP performed the main laboratory experiments, LD performed bioinformatic analyses, analyzed the data and coordinated the project, RM isolated and characterized the ZM3 strain, JB and MS identified and analyzed transposable elements, MS constructed mini-derivatives of plasmid pZM3H1, DB designed the project and supervised the work, LD and DB wrote the manuscript. All authors read and approved the final version of the manuscript.

## Supplementary Material

Additional file 1: Table S1. Description of ORFs located within plasmid pZM3H1 of *Halomonas* sp. ZM3. The table indicates characteristic features of distinguished ORFs, including their position, transcriptional orientation, the size of the encoded proteins, and their closest known homologs. (DOC 128 kb) Click here for file
