# Supplementary material for: Characterization of Halomonas sp. ZM3 isolated from the Zelazny Most post-flotation waste reservoir, with a special focus on its mobile DNA
Source: BMC Microbiol. 2013 Mar 14;13:59. doi: 10.1186/1471-2180-13-59 (PMC3606827; doi:10.1186/1471-2180-13-59)
Supplement: Additional file 1: Table S1. — Description of ORFs located within plasmid pZM3H1 of Halomonas sp. ZM3. The table indicates characteristic features of distinguished ORFs, including their position, transcriptional orientation, the size of the encoded proteins, and their closest known homologs. (DOC 128 kb) [file 1471-2180-13-59-S1.doc]

**Table S1.** Description of ORFs located within plasmid pZM3H1 of *Halomonas* sp. ZM3.

| ORF no. | **Coding region**  **(bp)** | **Strand** | **Protein size (aa)** | **Possible function** | **Best BLAST hits** | | |
| --- | --- | --- | --- | --- | --- | --- | --- |
|  |  |  |  |  | **% identity (aa)** | **Organism** | **GenBank accession no.** |
| 1 | 645-1979 | → | 444 | Replication initiation protein (RepA) | 49  (215/440) | uncultured bacterium (plasmid pRSB105) | ABI20460 |
| 2 | 2349-2918 | → | 189 | XRE family transcriptional regulator | 39  (30/76) | *Brevundimonas diminuta* ATCC  11568 | ZP_08268262 |
| 3 | 3134-3490 | → | 118 | Hypothetical protein, MerR family transcriptional regulator | 40  (27/67) | *Alteromonas* sp. SN2 | YP_004467511 |
| 4 | 3875-4912 | → | 345 | Hypothetical protein, Gp25 | 41  (148/359) | *Nitrosomonas sp.* Is79A3 | YP_004694670 |
| 5 | 5078-5698 | → | 206 | Hypothetical protein | - | No similarity found | - |
| 6 | 5957-6700 | → | 247 | Hypothetical protein | 48  (120/250) | *Nitrosomonas sp.* Is79A3 | YP_004694676 |
| 7 | 6718-7446 | → | 242 | Hypothetical protein | 51  (123/240) | *Nitrosomonas sp.* Is79A3 | YP_004694666 |
| 8 | 7693-8073 | ← | 126 | Hypothetical protein, membrane protein | 100  (126/126) | *Congregibacter litoralis* KT71 | ZP_01101118 |
| 9 | 8153-9229 | ← | 358 | Cointegrate resolution protein T (TnpT), chromosome segregation ATPases-like protein (KfrA) | 100  (358/358) | *Congregibacter litoralis* KT71 | ZP_01101119 |
| 10 | 9488-10393 | → | 301 | Cointegrate resolution protein S (TnpS), phage integrase | 100  (301/301) | *Congregibacter litoralis* KT71 | ZP_01101120 |
| 11 | 10416-10829 | ← | 137 | Co/Zn/Cd efflux system component (CzcD) | 99  (136/137) | *Congregibacter litoralis* KT71 | ZP_01101121 |
| 12 | 10932-11147 | ← | 71 | Co/Zn/Cd efflux system component (CzcD) | 99  (70/71) | *Congregibacter litoralis* KT71 | ZP_01101122 |
| 13 | 11348-11785 | ← | 145 | Hypothetical protein | 100  (145/145) | *Congregibacter litoralis* KT71 | ZP_01101123 |
| 14 | 11825-11947 | ← | 40 | Hypothetical protein | 100  (40/40) | *Congregibacter litoralis* KT71 | ZP_01101124 |
| 15 | 11974-12672 | ← | 232 | Beta-lactamase family protein | 100  (232/232) | *Congregibacter litoralis* KT71 | ZP_01101125 |
| 16 | 12886-13314 | ← | 135 | MerR family transcriptional regulator (MerR) | 100  (135/135) | *Congregibacter litoralis* KT71 | ZP_01101126 |
| 17 | 13388-13741 | → | 117 | Mercury ion transport protein (MerT) | 100  (117/117) | *Congregibacter litoralis* KT71 | ZP_01101127 |
| 18 | 13755-14075 | → | 106 | Mercuric transport protein periplasmic component (MerP) | 100  (106/106) | *Congregibacter litoralis* KT71 | ZP_01101128 |
| 19 | 14316-15719 | → | 467 | Mercuric reductase (MerA) | 97  (454/467) | *Congregibacter litoralis* KT71 | ZP_01101129 |
| 20 | 15733-16356 | → | 207 | Alkylmercury lyase (MerB) | 94  (194/207) | *Congregibacter litoralis* KT71 | ZP_01101130 |
| 21 | 16358-16759 | → | 125 | MerR family transcriptional regulator (MerD) | 98  (122/125) | *Congregibacter litoralis* KT71 | ZP_01101131 |
| 22 | 16752-17060 | → | 102 | Mercury resistance protein (MerE) | 79  (48/61) | *Congregibacter litoralis* KT71 | ZP_01101132 |
| 23 | 17124-17519 | → | 131 | Hypothetical protein, globin | 85  (112/131) | *Congregibacter litoralis* KT71 | ZP_01101135 |
| 24 | 17777-18055 | → | 92 | Hypothetical protein | 66  (61/92) | *Halomonas boliviensis* LC1 | ZP_09189875 |
| 25 | 18502-18774 | ← | 90 | Hypothetical protein | 43  (39/90) | *Thiobacillus denitrificans* ATCC 25259 | YP_314697 |
| 26 | 18771-19724 | ← | 317 | Phage integrase | 51  (160/312) | *Thiobacillus denitrificans* ATCC 25259 | YP_314696 |
| 27 | 20030-20347 | ← | 105 | Hypothetical protein | - | No similarity found | - |
| 28 | 20914-21216 | ← | 100 | XRE family transcriptional regulator, antitoxin of toxin-antitoxin system | 62  (62/100) | *Brenneria sp.* EniD312 | ZP_09017283 |
| 29 | 21226-21570 | ← | 114 | Gp49-like protein, toxin of toxin-antitoxin system | 68  (78/114) | *Xenorhabdus nematophila* ATCC  19061 (plasmid XNC1_p) | YP_003662458 |
| 30 | 22111-22356 | ← | 81 | Hypothetical protein | 48  (26/54) | *Salmonella sp.* 14  (plasmid p14-95A) | AFK89922 |
| 31 | 23138-23737 | → | 199 | Hypothetical protein, Heat shock protein C | 55  (90/164) | *Acidithiobacillus ferrivorans* SS3 | YP_004784374 |
| 32 | 24343-25743 | → | 466 | Mobilization protein (MobA) | 33  (112/335) | *Oceanimonas sp.* GK1 (plasmid pOCEGK02) | YP_005093727 |
| 33 | 25889-26161 | → | 90 | Hypothetical protein | 84  (53/63) | *Halomonas sp.* HAL1 | ZP_08958813 |
| 34 | 26416-27060 | → | 214 | Partition protein parA (ParA) | 56  (120/216) | *Pseudomonas syringae* pv. glycinea str. B076 | EFW77372 |
| 35 | 27092-27334 | → | 80 | Hypothetical protein, putative partition protein (ParB) | 47  (33/70) | *Pseudomonas syringae* pv. glycinea str. B076 | EFW77373 |
| 36 | 27436-27681 | → | 81 | Hypothetical protein | 42  (32/77) | *Azotobacter vinelandii* DJ | YP_002798969 |
| 37 | 27735-28136 | ← | 133 | Hypothetical protein | 31  (22/71) | *Desulfosporosinus acidiphilus* SJ4 | YP_006467616 |
| 38 | 28216-28497 | → | 93 | Prophage CP4-57 regulatory, AlpA-like protein | 32  (25/79) | *Pseudomonas syringae* pv. glycinea str. B076 | EFW81920 |
| 39 | 28537-29079 | → | 180 | Hypothetical protein | - | No similarity found | - |
| 40 | 29076-29351 | → | 91 | Hypothetical protein | 71  (67/87) | *Halomonas* sp. HAL1 | ZP_08959101 |
| 41 | 29631-30974 | → | 447 | Phage integrase | 33  (137/416) | *Hahella chejuensis* KCTC 2396 | YP_431950 |
| 42 | 31137-31370 | → | 77 | Hypothetical protein, MerR family transcriptional regulator | 41  (29/71) | *Alteromonas* sp. SN2 | YP_004467511 |
